# Theranostic potential of a novel aptamer specifically targeting HER2 in breast cancer cells

**DOI:** 10.55730/1300-0152.2680

**Published:** 2024-02-01

**Authors:** Fulya KÜÇÜKCANKURT, Samet UÇAK, Nedret ALTIOK

**Affiliations:** 1 Department of Medical Biology, School of Medicine, Altinbas University, İstanbul, Turkiye; 2 Department of Medical Biology, School of Medicine, Istanbul Aydin University, İstanbul, Turkiye; 3 Department of Pharmacology and Medical Pharmacology, School of Medicine, Istinye University, İstanbul, Turkiye

**Keywords:** HER2, breast cancer, cell-SELEX, aptamer, targeted therapy, optical imaging

## Abstract

**Background/aim:**

The overexpression of HER2 is correlated with poorer outcomes and therapeutic resistance in breast cancer patients. While HER2-targeted therapies have shown improvement, prognosis remains poor for HER2-positive breast cancer patients, and these treatments have limitations. Therefore, it is crucial to explore effective molecular strategies for early detection and treatment of HER2-positive breast cancers.

**Materials and methods:**

In this study, we employed the cell-SELEX method to generate a selective aptamer capable of recognizing HER2 in its native conformation within breast cancer cells, for theranostic applications. Utilizing an adherent cell-SELEX approach, we developed and explored a DNA aptamer, named HMAP7, which can specifically target HER2 in the MDA-MB-453 and SK-BR-3 human breast cancer cell lines. After sequencing, the binding affinities of 10 candidate aptamers to HER2 receptors were evaluated by measuring fluorescence intensities within intact cells using near-infrared optical imaging. The dissociation constant of HMAP7 was determined to be in the nanomolar range in both cell lines.

**Results:**

The cell-SELEX-derived aptamer sequence, HMAP7 (41-mer), exhibited the highest binding affinity and specificity for HER2. HMAP7 was rapidly internalized into breast cancer cells overexpressing HER2 but showed no uptake in the HER2 receptor-deficient breast cancer cell line MDA-MB-231. Moreover, HMAP7 demonstrated remarkable selectivity for HER2, rendering it suitable for use in complex biological systems.

**Conclusions:**

Our findings suggest that the novel DNA aptamer HMAP7 holds promise for both therapeutic and diagnostic applications, enabling selective delivery of therapeutic agents or imaging of HER2-positive breast tumors.

## 1. Introduction

Approximately 20% of human breast cancers are characterized by the overexpression of human epidermal growth factor receptor 2 (HER2), which regulates cellular survival and proliferation. HER2-directed monoclonal antibodies (such as pertuzumab and trastuzumab) along with targeted cytotoxic drug delivery through HER2 receptor antibody-drug conjugates (such as trastuzumab deruxtecan and trastuzumab emtansine) have demonstrated some success in targeting HER2 molecules (Ghosh et al., 2011; Krop and Winer, 2014; Gampenrieder et al., 2020; Kunte et al., 2020). However, these drug therapies have significant limitations. Apart from notable adverse drug reactions, they also lead to the rapid development of drug resistance in HER2-positive breast cancer patients (Dent et al., 2013; Kümler et al., 2014; Kumar et al., 2020). These drawbacks could be overcome with the development of selective agents targeting HER2 at the molecular level. When antibodies are employed as imaging tools, they are poorly tolerated due to their native antigenicity and large size, which limits their ability to penetrate tumors with high degrees of compaction. Consequently, over the past decade, many aptamers designed to target cell surface biomarkers have emerged as high-affinity ligands capable of overcoming the limitations of antibodies for both therapeutic and diagnostic applications (Keefe et al., 2010; Li and Lee, 2019; Zhang et al., 2019).

Aptamers, which are short synthetic single-stranded RNA or DNA molecules typically consisting of 20–60 oligonucleotides, exhibit high affinity for binding to various molecular targets, including small molecules, nucleic acids, tissues, cells, and proteins (Kumar et al., 2020). They possess several advantageous properties, being chemically stable, modifiable, nonimmunogenic, and nontoxic, with good tissue penetration, facilitating their utilization in biosensing, diagnostics, and therapeutics (Ning et al., 2020; Li et al., 2022; Liu et al., 2023). The method for producing aptamers, known as Systematic Evolution of Ligands by EXponential enrichment (SELEX), particularly when utilizing a cell line, is referred to as cell-SELEX (Camorani et al., 2020; Zhong et al., 2020). This method can efficiently isolate aptamers capable of binding to cell surface proteins in their original configurations, as living cells serve as the specific targets of selection (Lin et al., 2021). Due to their attractive properties, numerous research groups have developed aptamers targeting HER2 either as single agents or in combination with multiple drugs and nanomaterials for treating HER2-positive breast cancers (Jiang et al., 2017; Zhu et al., 2017; Liu et al., 2018). Recent reports have also highlighted aptamer applications for imaging and diagnosis, suggesting their potential as valuable tools for diagnosing and imaging various cancers (Soontornworajit and Wang, 2011; Zhang et al., 2019; Ning et al., 2020; Zhong et al., 2020; Cong et al., 2023). Despite the advantages aptamers offer over antibodies, numerous challenges must be addressed before aptamer-based therapeutics or imaging tools for various cancer types can be developed (Ni et al., 2017; Mo et al., 2022; Wang et al., 2022; García Melián et al., 2023).

In this study, we employed the adherent whole Cell-SELEX method to generate a novel DNA aptamer targeting HER2 in HER2-overexpressing breast cancer cells. The breast cancer cell lines MDA-MB-453 and SKBR are HER2-positive and estrogen receptor/progesterone receptor-negative, while MDA-MB-231 is triple-negative regarding Her-2/neu protein, estrogen, and progesterone expression (Holliday and Spairs, 2011).

The developed HER2-targeted DNA aptamer was labeled with the near-infrared (NIR) fluorescent dye IRD800CW, enabling its use for optical imaging or targeted HER2 therapy in patients with HER2-overexpressed cancers.

## 2. Materials and methods

### 2.1. Cell line and culture conditions

The human metastatic breast cancer cell lines MDA-MB-453 (HTB-131), SK-BR-3 (HTB30), and MDA-MB-231 (HTB-26) were obtained from ATCC (American Type Culture Collection). These cell lines were cultured in DMEM/F12 media (Gibco) supplemented with 10% fetal bovine serum (Gibco), 1% penicillin-streptomycin (10,000 U/mL) (Gibco), and 1% L-glutamine (200 mM) (Gibco) in a 5% CO_2_ atmosphere at 37 °C. The culture medium was refreshed every other day, and subculturing was performed according to the manufacturer’s protocol. For experimentation, primary antibodies against HER2 (2165S), HER3 (12708S), and EGFR (4267S) were obtained from Cell Signaling Technology, while anti-ErbB2 Affibody-Biotin was acquired from Abcam. Additionally, a 680RD-labeled secondary antibody (LI-COR) and CellTag 700 (LI-COR) were utilized.

### 2.2. Cell-SELEX procedure

The cell-SELEX procedure started with the preparation of a nucleic acid library. Initially, a large random DNA pool was incubated with the target molecule. To distinguish oligonucleotides that fold and form complex structures with the target from nonbinding types, target molecules were blocked on a carrier during selections. The nucleic acid pool containing target-bound sequences was then amplified using PCR (Komarova and Kuznetsov, 2019). The PCR products were utilized to regenerate single-stranded DNA (ssDNA), creating a fresh oligonucleotide pool enriched with more target-binding nucleotides. These steps of binding, separation, amplification, and pool regeneration were repeated until the nucleic acid pool comprised a sufficient number of target-binding sequences. The enriched pool was subsequently subjected to sequencing and subsequent analysis to identify potential aptamer candidates (Komarova and Kuznetsov, 2019; Kohlberger and Gadermaier, 2022).

**Figure 1:**
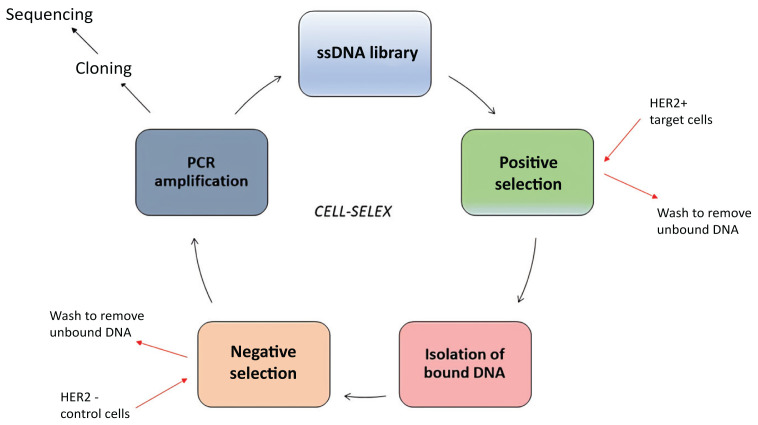
Aptamer selection with cell-SELEX method. A total of 15 selection rounds were applied to enrich DNA aptamer sequences that bind to HER2-overexpressing live breast cancer cell lines SK-BR-3 and MDA-MB-453. MDA-MB-231 cells were used for negative selection.

To isolate aptamers specifically targeting HER2-positive breast cancer cells, we selected MDA-MB-453 and SK-BR-3 cells as targets and mediated negative selection using MDA-MB-231 cells. Figure 1 illustrates the schematic model of the cell-SELEX method employed for aptamer selection.

The ssDNA SELEX library used for selection comprised a 41-nucleotide random region bounded by fixed regions containing primer sites for the amplification reaction (Table 1). Although 85 bp oligonucleotides were employed in the SELEX cycles, only the random region oligonucleotides were tested during the characterization of aptamer candidates.

Each cell line was incubated with a 30 nM SELEX library at 37 °C for 1 h in cell culture media. Subsequently, target-bound library sequences were amplified using PCR with biotinylated primers (Table 1).

The PCR product, comprising double-stranded DNA (dsDNA), was then incubated with streptavidin-coated magnetic beads at a concentration of 0.4 mg/mL for 2 h. The dsDNA bound to the magnetic beads was denatured under alkaline conditions (0.1 M NaOH), resulting in the retrieval of biotinylated ssDNA. This biotinylated ssDNA was used for aptamer selection.

**Figure 2:**
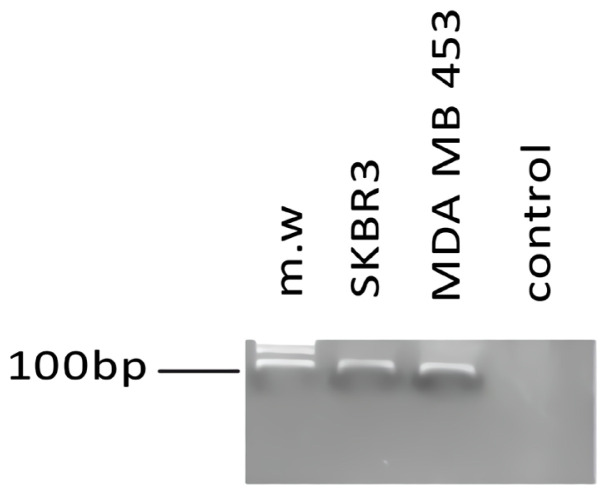
The amplification products from PCR analysis. HMAP7 bands were obtained from SK-BR-3 and MDA-MB-453 cells, but not from negative control MDA-MB-231 cells. Molecular weight (mw) (NEB Biotech Inc., 2-log DNA ladder), and samples were run on 3% agarose gel electrophoresis using ethidium bromide staining (Enduro GDS gel documentation system, Labnet Int. Inc.).

After the first SELEX round, ssDNA obtained from MDA-MB-231 cells was incubated with MDA-MB-453 and SK-BR-3 cells for negative selection to eliminate the sequences binding to surface antigens other than HER2. The resulting aptamer pool was then utilized in subsequent rounds of selection, with the entire process repeated. A total of 15 selection rounds were performed. The enrichment of HER2-specific aptamers within a particular aptamer pool was analyzed with agarose gel electrophoresis (Figure 2).PCR amplification was performed under the following conditions: 2 min at 94 °C; 15 cycles of 10 s at 94 °C, 10 s at 62 °C, 10 s at 72 °C; 2 min at 72 °C. The selected aptamer pools were separated using the TOPO TA Cloning Kit for Sequencing (Invitrogen, Thermo Scientific), and the positive transformants were analyzed via Sanger DNA sequencing using CLC Main Workbench 7.1 (Qiagen Bioinformatics).

### 2.3. Cell binding assay

Live cells (1 × 10^4^) were seeded in 96-well plates and incubated with various concentrations (ranging from 0.01 to 10 μM) of IRD 800-labeled aptamers at the 5′ end. This incubation took place in growth medium containing 5% FBS at 37 °C for 1 h. All oligos were purchased from Integrated DNA Technologies, Inc. (Coralville, IA).

Following the incubation period, the medium was aspirated, and the cells were washed. Fluorescence intensities were then quantified in the 800 nm channel of the LI-COR Odyssey Infrared Imaging System. Subsequently, the cells were fixed and permeabilized using a Triton washing solution, and CellTag 700 (LI-COR) stain was added. The cells were further incubated for 1 h to correct for variations in cell number from well to well. After washing, the CellTag 700 stain was detected in the 700 nm channel of the LI-COR Odyssey Infrared Imaging System. SigmaPlot software (version 14.5) was used for data plotting and curve fitting to estimate the dissociation constants (Kd), using the equation Y = BmaxX / (Kd + X), where Bmax represents maximum fluorescence intensity, Y represents fluorescence intensity at each concentration, and X represents aptamer concentration (Lin et al.,2020).

### 2.4. On-cell western plate-based assay

The treated, fixed, permeabilized, and blocked cells were incubated with primary antibodies overnight in a blocking buffer, as specified. After washing, secondary antibodies labeled with either 800CW or 680RD (LI-COR) were added and incubated for 1 h. Subsequently, the 96-well plates were washed. CellTag 700 (LI-COR) staining was conducted simultaneously with 800CW-conjugated secondary antibody for normalization of cell numbers. After washing, the plates were scanned in either the 800 nm or 700 nm channels of the LI-COR Odyssey Infrared Imaging System.

### 2.5. NIR-labeled capture immunoassay for targeted optical imaging

Streptavidin-coated 96-well plates were washed and incubated with biotinylated anti-HER2 affibody, serving as the capture antibody, for 2 h at room temperature. Following this incubation, the HMAP7-IRD800-treated cell lysate was added to the plate and incubated for 1 h. After incubation, the plate was washed and scanned in the 800 nm channel of the LI-COR Odyssey Infrared Imaging System. Subsequently, the plates were fixed and incubated with anti-HER2 primary antibody overnight in blocking buffer, as specified. After washing, a 680RD-labeled secondary antibody was added and incubated for 1 h. Following another round of washing, the plates were scanned in the 700 nm channel of the LI-COR Odyssey Infrared Imaging System.

## 3. Results

### 3.1 Selection of DNA aptamers against HER2-overexpressing cells

To select aptamers targeting HER2 proteins associated with the cell surface in their inherent condition, we applied cell-SELEX using HER2-overexpressing live MDA-MB-453 and SK-BR-3 breast cancer cell lines as targets. For negative selection, we utilized MDA-MB-231 cells. The main benefit of using adherent cells lies in their ability to be easily washed, facilitating the removal of nonbound sequences as well as dead or floating cells. A total of 15 subsequent rounds of selection were conducted before the Cell-SELEX process was concluded. Sanger sequencing analysis revealed 3 random oligo (N41) sequences from SK-BR-3 cells (designated as “K”) and 7 random oligo (N41) sequences from MDA-MB-453 cells (designated as “M”), which matched the sequences present in the initial library (Table 2).

### 3.2. The expression levels of HER2, HER3, and EGFR in breast cancer cell lines

**Figure 3:**
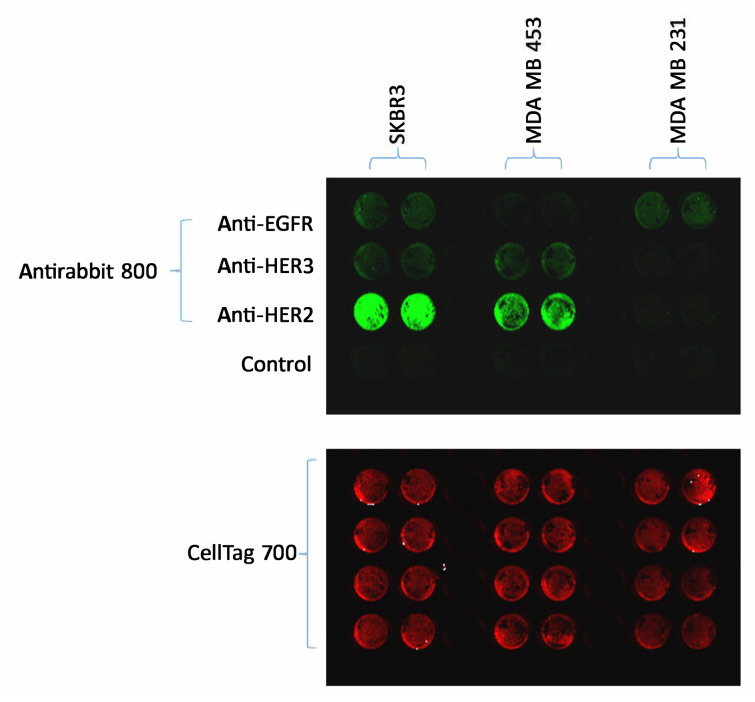
Expression levels of HER2, HER3, and EGFR on breast cancer cells. Receptor protein levels in MDA-MB-453, SK-BR-3, and MDA-MB-231 cells were detected by on-cell western method using anti-HER2, anti-HER3, and anti-EGFR primary antibodies, then antirabbit 800 (green) conjugated secondary antibody and CellTag 700 (red) stain for normalization of cell number. The plates were scanned in the 800 or 700 nm channels of LI-COR Odyssey Infrared Imaging System.

To analyze the binding characteristics of the 10 selected aptamers to HER2 receptors, we initially determined the expression levels of HER2 receptors on HER2-positive MDA-MB-453 and SK-BR-3, as well as HER2-negative MDA-MB-231 human breast cancer cell lines. This analysis was conducted using the LI-COR Odyssey Infrared Imaging System (Figure 3). Our results demonstrated that SK-BR-3 cells express HER2, HER3, and EGFR, MDA-MB-453 cells express HER2 and HER3, while MDA-MB-231 cells only possess EGFR.

### 3.3. Concentration-dependent receptor-based binding of IRD800-labeled HMAP7 to HER2 overexpressing cells

**Figure 4:**
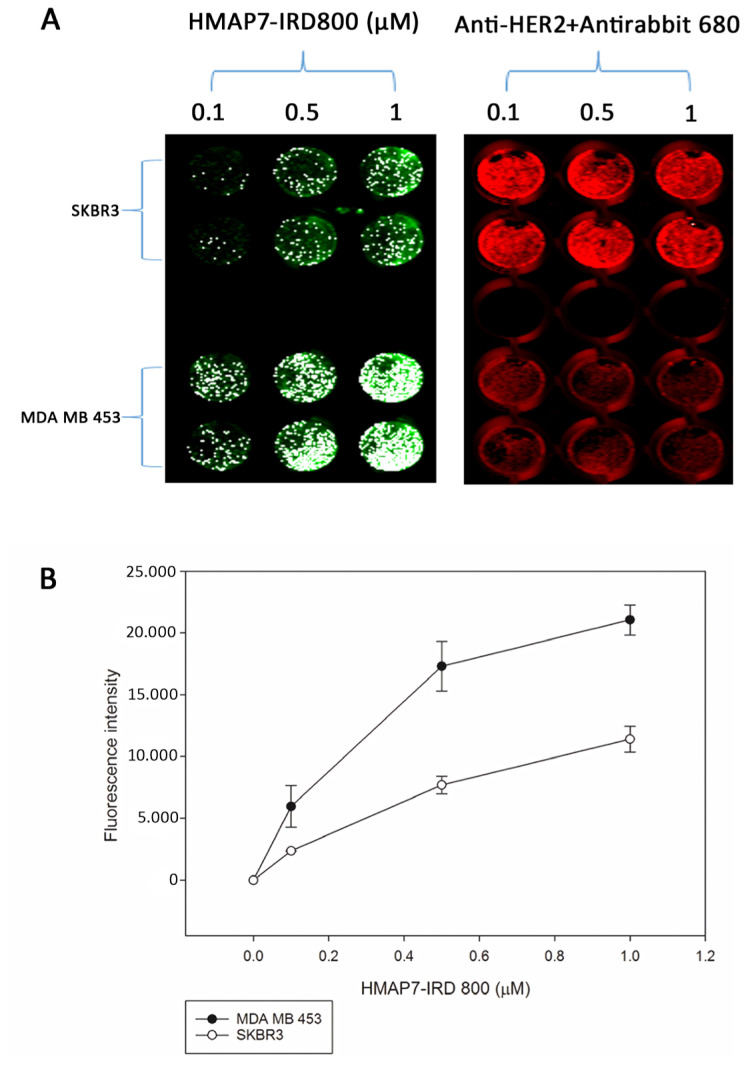
Concentration-dependent binding of HMAP7 to HER2 overexpressing breast cancer cells. **(A)** MDA-MB-453 and SK-BR-3 cells were incubated with increasing concentrations of IRD800-labelled HMAP7 (green) for 1 h. Subsequently, the fluorescence intensities were detected by Infrared Imaging System and used to calculate the dissociation constant Kd value of HMAP7. Anti-HER2 primary and 680RD–labeled (red) secondary antibodies were used to detect HER2 levels. **(B)** The graph of HMAP7-IRD 800 concentration against fluorescence intensity was plotted. Error bars were calculated as standard error.

Next, we assessed the efficiency of binding and internalization of NIR fluorescent dye IRD800CW-labeled aptamers to HER2-positive breast cancer cells. Live cells (1 × 10^4^) were seeded in 96-well plates and incubated with various concentrations (ranging from 0.01 to 10 μM) of IRD800-labeled aptamers at the 5′ end in medium containing 5% FBS at 37 °C or 4 °C for 1 h. Fluorescence intensity was then quantified using an NIR fluorescence imager. All 10 selected aptamers, as listed in Table 2, were found to bind to the HER2-positive cells, but aptamer HMAP7 had the highest calculated binding affinity in HER2-overexpressing MDA-MB-453 and SK-BR-3 cells. However, none of these 10 aptamers exhibited detectable binding to HER2-negative MDA-MB-231 cells. Additionally, in our control experiments, we utilized a mutant HMAP7 sequence (Table 2) and observed no binding to MDA-MB-453 and SK-BR-3 cells. Cells were incubated with increasing concentrations of IRD800-labeled aptamers for 1 h, and the fluorescence intensities were then detected using an Infrared Imaging System. These intensities were used to calculate the dissociation constant (Kd) of HMAP7 in HER2-overexpressing MDA-MB-453 and SK-BR-3 cells. The Kd of HMAP7 was determined to be 0.260 ± 0.04 μM in MDA-MB-453 cells and 0.350 ± 0.07 μM in SK-BR-3 cells (Figures 4A and 4B).

The signal intensities of IRD800-labeled HMAP7 binding to HER2-positive cells exhibited a gradual increase from 15 min to 6 h of incubation. Following a 1-h incubation at 37 °C and removal of excess conjugates, numerous green spotted patterns were observed within the target cells, indicating an effective targeting mechanism of the aptamers to the HER2-positive cells. Interestingly, the binding affinity of HMAP7 to SK-BR-3 cells was lower than that to MDA-MB-453 cells, possibly due to variations in the dimerization partners of HER2 in these cells. The higher binding affinity of IRD800-labeled HMAP7 to MDA-MB-453 cells may suggest that HER2/HER3 is the preferred heterodimer for HMAP7 compared to SK-BR-3 cells, which possess EGFR.

### 3.4. The comparison of the receptor-based binding specificities of selected and reported aptamers to HER2 overexpressing cells

**Figure 5:**
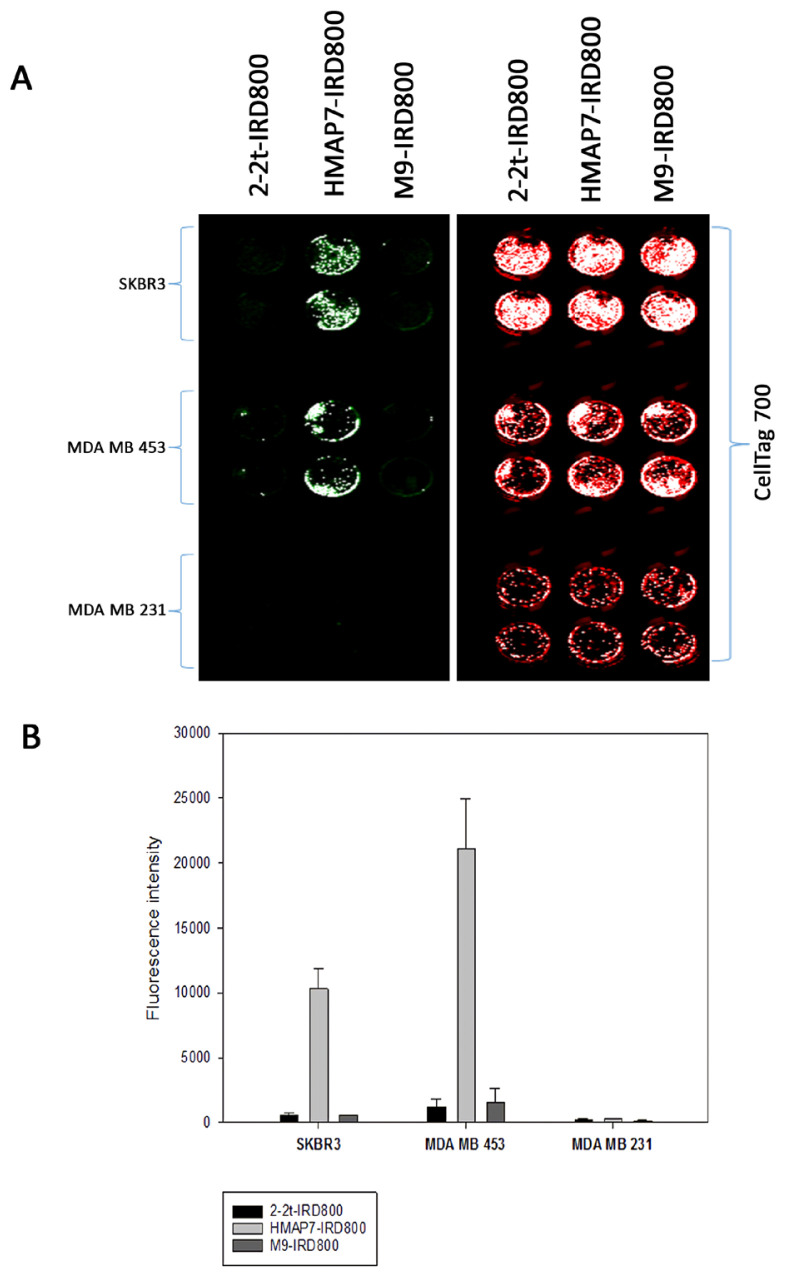
The comparison of the receptor-based binding specificities of selected (HMAP7 and M9) and reported (2-2t) aptamers. **(A)** HER2-overexpressing MDA-MB-453 and SK-BR-3, and HER2-negative MDA-MB-231 cells were incubated with IRD 800-labeled (green) HMAP7, M9 or 2-2t aptamers for 1 h. Subsequently, CellTag 700 (red) applied on-cell western assay. Mean fluorescence intensities were detected by infrared imaging and plotted with standard error as shown **(B)**.

A trimeric HER2-targeting DNA aptamer, 2-2(t), previously reported in human gastric cancer cells (Mahlknecht et al., 2013), and one of our selected 10 aptamers with the lowest affinity to HER2 (M9), were utilized for comparison with HMAP7. All aptamers were used at a concentration of 1 μM. As illustrated in Figures 5A and 5B, the fluorescence binding signal of 2-2(t) was approximately 17 times lower than that of HMAP7 in both MDA-MB-453 and SK-BR-3 cells. This discrepancy may be attributed to variations in the conformational properties of the aptamers or the dimerization characteristics of the receptors in gastric and breast cancer cells. Notably, none of these IRD800-labeled aptamers exhibited binding to HER2-negative MDA-MB-231 cells.

### 3.5. Receptor-based binding and competition assay

**Figure 6:**
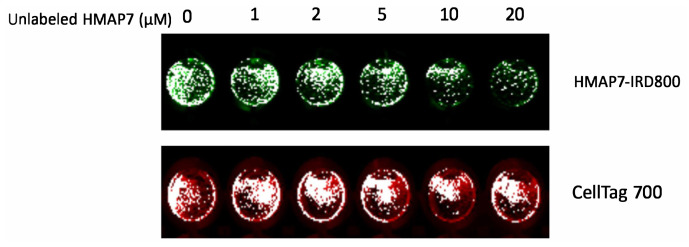
Receptor-based binding and competition assay. Binding of HMAP7-IRD800 (green) to MDA-MB-453 cells was almost completely inhibited by 20 μM of unlabeled HMAP7. CellTag700 (red) staining was carried out for cell number normalization. The plates were scanned in the 800 or 700 nm channels of LI-COR Odyssey Infrared Imaging System.

For competitive binding analysis (Figure 6), unlabeled HMAP7 aptamer ranging from 1 to 20 μM (up to a 20-fold molar excess) was preincubated with MDA-MB-453 cells for 15 min at 37 °C. Subsequently, 1 μM of IRD800-labeled HMAP7 aptamer was added and further incubated for 1 hour. The competitive cell binding assay demonstrated that the binding of IRD800-labeled HMAP7 to cells was almost completely inhibited by 20 μM of unlabeled HMAP7.

Thus, the selectivity of HMAP7 for HER2 receptors was demonstrated by two key observations. Firstly, there was a lack of binding to HER2-negative MDA-MB-231 cells. Secondly, a dose-dependent decrease in the binding of labeled aptamer to HER2-positive MDA-MB-453 cells was observed in the presence of unlabeled aptamer.

### 3.6. NIR-labeled capture immunoassay for targeted optical imaging

**Figure 7:**
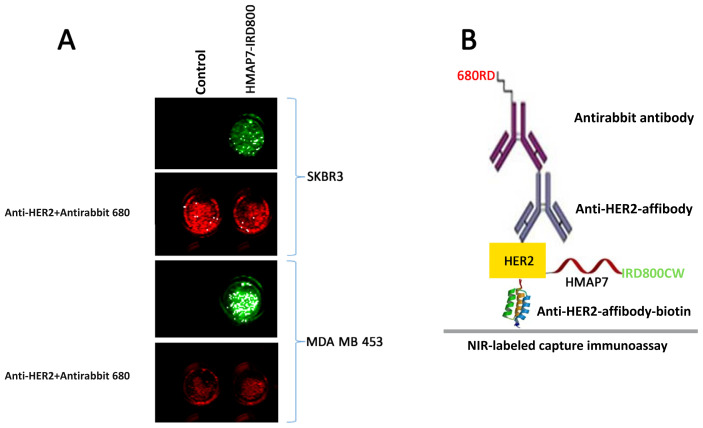
**(A)** NIR-labeled capture immunoassay for targeted optical imaging. HMAP7-IRD800-treated (green) or IRD800-treated (control) MDA-MB-453 cell lysates were incubated with the streptavidin-coated 96-well plate pretreated with biotinylated anti-HER2-affibody. Then anti-HER2 primary antibody was used and detected with antirabbit 680RD secondary antibody (red) and scanned in the 800 nm and 700 nm channels of LI-COR Odyssey Infrared Imaging System. **(B)** Schematic diagram of NIR-labeled capture immunoassay.

To further investigate whether HMAP7 binds specifically to HER2 receptors, HMAP7-IRD800-treated cell lysates were incubated with streptavidin-coated 96-well plates pretreated with biotinylated anti-HER2-affibody as the capture antibody. Subsequently, an anti-HER2 primary antibody was used, followed by antirabbit 680RD, and the samples were scanned in the 800 and 700 nm channels of the LI-COR Odyssey Infrared Imaging System. The detection of HMAP7-IRD800 in HER2-overexpressing MDA-MB-453 and SK-BR-3 cells (Figure 7A) with similar affinities observed in cell binding assays indicates the specific binding of HMAP7 to HER2. The schematic diagram of NIR-labeled capture immunoassays is presented in Figure 7B.

## 4. Discussion

In this study, we have developed a novel HER2-targeting DNA aptamer, HMAP7, which, when labeled with the NIR fluorescent dye IRD800CW, can serve as an optical imaging agent in HER2-overexpressed cancers. Our method offers advantages over traditional antibody labeling techniques because HMAP7-IRD800 accumulates in HER2 overexpressing cancer cells, enabling noninvasive and reversible cell labeling for tumor visualization using a NIR camera system.

Aptamers are emerging as highly promising candidates for selectively targeting tumor cells (Camorani et al.,2020). Their high stability, targeted effect, low immunogenicity, and low molecular weight make them attractive for cancer diagnosis and treatment (Zhou et al., 2017; Zhang et al., 2019; Ning et al., 2020).

Aptamers are typically produced using the SELEX method. While SELEX traditionally employs target proteins, utilizing living cells as selection targets allows for the selective targeting of proteins in different cell types. Through cell-SELEX, aptamers are isolated from random oligonucleotide libraries with multiple rounds of selection against whole cells in culture, thereby enabling the elimination of off-target cells (Camorani et al., 2020; Zhong et al., 2020).

In this study, we employed a cell-SELEX method to accurately identify HER2-positive breast cancer cells, utilizing Sanger sequencing to determine a panel of DNA aptamers that bind to HER2 receptors on target cells with an equilibrium dissociation constant (Kd) in the nanomolar range. These cell binding assays were conducted on live cells in growth medium, allowing the conformation of HER2 receptors to closely mimic biological functions for kinetic measurements. Notably, we demonstrated that the selected aptamer HMAP7 exhibited the highest potential to discriminate between HER2-positive and -negative breast cancer cells, suggesting its potential for specific targeting of HER2-overexpressing cancer cells.

The activation of HER2 receptors can be induced by hetero- or homodimerization with HER2 (Hsu and Hung, 2016) or other members of the ERbB/HER family, such as HER3 or EGFR, in both normal and tumor cells (Altiok et al., 1995, Gandullo Sánchez et al.,2022). Each breast cancer cell line used in our study exhibits a distinct expression profile of various HER receptors (Maadi et al.,2018), which could significantly impact the binding preference of our HER2 DNA aptamer HMAP7. Our findings revealed that, in addition to homodimerization of HER2/HER2, in SK-BR-3 cells HER2/HER3 or HER2/EGFR, and in MDA-MB-453 cells HER2/HER3 heterodimerizations are possible. The higher binding affinity of IRD800-labeled HMAP7 to MDA-MB-453 cells suggests that HER2/HER3 is the preferred heterodimer for HMAP7 compared to SK-BR-3 cells, which predominantly possess EGFR, potentially leading to diminished binding of HMAP7 to HER2 in the SK-BR-3 cell line. Moreover, IRD800-labeled HMAP7 was not taken up by HER2-negative MDA-MB-231 cells after the same duration of incubation in several experiments.

Before selecting our aptamers, we tested some of the reported sequences of oligonucleotides. However, when we labeled these reported aptamers (Mahlknecht et al., 2013) with NIR dye, we could not detect any specific binding or, as shown in Figure 5, we observed a very weak binding to HER2 in HER2-positive breast cancer cell lines compared to our selected aptamer HMAP7 in our experimental model.Similar to the HMAP7 aptamer we have developed, aptamers targeting surface receptors can be conjugated with various agents such as chemotherapeutics, small molecule inhibitors, nanoparticles, siRNAs, or peptides to increase their clinical efficacy in targeted drug delivery for cancer therapy (Lyu et al., 2016; Maimaitiyiming et al., 2019; Ning et al., 2020; Mo et al., 2022; Liu et al., 2023). Furthermore, regarding its therapeutic efficacy, fluorophore-labeled HER2-aptamers could serve as specific tools for noninvasive molecular imaging of HER2-positive breast cancer cells. Although the aptamer generated in this study demonstrates remarkable selectivity for HER2 receptors on the surface of HER2-positive breast cancer cells, further analyses are necessary to improve the hallmarks of the DNA aptamer HMAP7 through chemical modifications (Ni et al., 2017; Camorani et al., 2020) to increase its nuclease resistance, and to assess its efficacy in vivo.

## 5. Conclusion

Using cell-SELEX technology, we have identified aptamer HMAP7 with a high affinity against HER2 overexpressing breast cancer cells. Aptamer HMAP7 demonstrates the ability to distinguish HER2-positive breast cancer cells from other cancer cell types with remarkable specificity. This high specificity renders aptamer HMAP7 an optimal choice for noninvasive tumor imaging. Consequently, aptamer HMAP7 holds enormous potential as an imaging agent or for delivering therapeutics to improve the diagnosis and treatment of HER2-positive breast cancer.

## Figures and Tables

**Table 1:** DNA library sequence and PCR primers used in SELEX*.

Name	Sequence
SELEX library	5′TAGGGAAGAGAAGGACATATGAT–N_41_-TCAAGTGGTCATGTACTAGTCAA3′
Forward primer	5′TAGGGAAGAGAAGGACATATGAT 3′
Reverse primer	5′TCAAGTGGTCATGTACTAGTCAA 3′
Biotinylated forward primer	5′Biotin-TAGGGAAGAGAAGGACATATGAT 3′

**Notes:**

* http://www.sumobrain.com/patents/wipo/Aptamers-purifying-quantifying-gelsolinits/WO2016056028A2.html

**Table 2:** Sequences of identified aptamers and mutated HMAP7.

Name	DNA sequence
M1	5′- ACCATCACGCCGACGTTTACCCACACAGCCAGAATCCCCCC-3′
M7 (HMAP7)*	5′- CCGCCCAAACACACGTAAACAGATGAGGAGCCGACACTGGG-3′
M9	5′- CCCACGATCTTCGTACCGGTCAATGCTTCAGCCTTGCGACG 3′
M17	5′- CACCCAAACTTGGTCTGGATACCCCTTATGCGCGTAGTTAC 3′
M22	5′- GCGCCGGCACCCCCCCGAGAATACTCCCACCACGTACACTT 3′
M23	5′- CTCTTCGGGTGGGTGCGGGGTCCGAGTACTGTCTCACAGTG 3′
M27	5′- CCAATGTATGGGCGGTCGGTGGATACACGTCTTAGCGTCGT 3′
K10	5′- ACCAATCGCTCGTCATCACATAAAGAACCCCTCGAACCCCG-3′
K12	5′- TGGTCAATAATGTCCGGTAAGAGGCGCACAACTGGTTCGGT 3′
K15	5′- GCCGCCGAAGAACTCTCAGCACGACCAGCCACCCCACGAGG-3′
Mutant HMAP7	5′-GCAGCAGTGTGAGGGCAGCAGTGTGAGGGCAGCAGTGTGGG-3′

**Abbreviations:**

* HMAP7; HER MDA-MB-453 APTAMER M7
